# Prevalence of human alveolar echinococcosis in China: a systematic review and meta-analysis

**DOI:** 10.1186/s12889-020-08989-8

**Published:** 2020-07-14

**Authors:** Xuanzhuo Wang, Guodong Dai, Min Li, Wanzhong Jia, Zhongmin Guo, Jiahai Lu

**Affiliations:** 1 School of Public Health, Sun Yat-sen University, Guangzhou, Guangdong Province China; 2 State Key Laboratory of Veterinary Etiological Biology, Key Laboratory of Veterinary Parasitology of Gansu Province, Lanzhou Veterinary Research Institute, Chinese Academy of Agricultural Sciences, Lanzhou, Gansu Province China; 3 Experimental Animal Center, Sun Yat-sen University, Guangzhou, Guangdong Province China; 4 Key Laboratory for Tropical Diseases Control of Ministry of Education, Sun Yat-sen University, Guangzhou, Guangdong Province China; 5 One Health Research Centre, School of Public Health, Sun Yat-sen University, Guangzhou, Guangdong Province China

**Keywords:** Human alveolar echinococcosis prevalence, Systematic review and meta-analysis, One health, Disease control

## Abstract

**Background:**

Human alveolar echinococcosis (HAE), caused by the larvae of *Echinococcus multilocularis,* is a severe parasitic disease that is a major public health concern. New HAE cases in China account for 91% of the global HAE burden every year. Although there are a few studies and systematic reviews (SRs) on the prevalence of HAE in China, trends in the prevalence have not been estimated. This study aims to describe the overall variation in the trend of HAE prevalence in China, and provide evidence for preventive measures in the future.

**Methods:**

Thirty-five eligible studies were retrieved from PubMed, Web of Science, EMBASE, CNKI, Wanfang Data, and VIP, and included in the SR and meta-analysis. An adjusted Agency for Healthcare Research and Quality checklist was used to evaluate study quality. The arcsine transformation was used to adjust the individual reported prevalence, and the pooled HAE prevalence was calculated. Heterogeneity was evaluated using the chi-square test and *I*^*2*^ statistic. Forest plots were generated for the meta-analysis, and publication bias of the studies was assessed using the Egger’s test and funnel plots. We conducted subgroup analyses, sensitivity analyses, and meta-regression analyses to analyze the source of heterogeneity and factors potentially influencing the prevalence of HAE.

**Results:**

The meta-analysis indicated that the pooled HAE prevalence in China was 0.96% (95% CI: 0.71 to 1.25%). Factors potentially influencing HAE prevalence were female sex (OR = 1.60, 95% CI: 1.35 to 1.91, *P*<0.01), being ≥30 years old (OR = 4.72, 95% CI: 2.29 to 9.75, *P*<0.01), and being farmers and/or herdsmen (OR = 2.54, 95% CI: 1.60 to 4.02, *P*<0.01). The results of the meta-regression analysis (R^2^ = 38.11%, *P* < 0.01) indicated that HAE prevalence is on a downward trend.

**Conclusions:**

HAE prevalence has decreased over time and maintained low levels after 2005 in China. This decline was influenced by the utilization of One Health strategies as intervention measures. Therefore, these One Health strategies should be used as references to formulate future programs for HAE control. More high-quality epidemiological investigations and surveillance programs should be conducted in order to improve HAE control in the future.

## Background

Alveolar echinococcosis (AE) is a severe zoonosis caused by the larvae of *Echinococcus multilocularis* that has an adverse impact on human and animal health [1]. Parasite eggs are excreted through the feces of the definitive hosts, which are usually foxes and dogs. Once the intermediate hosts such as rodents and humans ingest the eggs, oncospheres released from the eggs under the action of gastrointestinal digestion go on to form metacestodes in the liver or other organs. A large number of protoscolices (PSCs) are usually generated by metacestodes through asexual reproduction. After the definitive hosts ingest the viscera that include the metacestodes, the PSCs attach to the definitive hosts’ intestine wall and develop into adult worms to complete the life cycle [2]. As one of the intermediate hosts, humans can contract hydatid disease through contaminated food or water, with the liver primarily being the infected organ. If not treated in time, the disease will lead to jaundice, cirrhosis, and other clinical symptoms, which can result in liver failure or even death. The case fatality of untreated human alveolar echinococcosis (HAE) patients or inadequately treated patients is 90% at 10–15 years after diagnosis [3, 4]. Hence, HAE is also known as “the parasitic cancer” [5].

HAE has become a global threat to public health [6–8]. There are 18,235 new cases of HAE every year in the world, with the disease burden reaching 666,433 disability-adjusted life years (DALYs) [9, 10]. In the past, it was generally believed that HAE cases were mainly distributed across the northern hemisphere, especially in Asia [11]. However, in recent years, new cases of HAE have been found in some European countries; the incidence of HAE has doubled in France and Germany [12–14]. Some regions of Canada have also reported cases of HAE [15]. China is one of the countries seriously affected by HAE, accounting for 91% of the global burden of new HAE cases every year [10]. The prevalence increased to 9.43% in Banma County, Qinghai in 2014 [16] and 3028 cases were reported in Shiqu County, Sichuan between 2015 and 2017 in China [17]. HAE has also become a burden in endemic regions and has constrained the development of animal husbandry in China [18].

Although a few studies and systematic reviews (SRs) have explored HAE in China, the trend in the variation of HAE prevalence remains unclear. Therefore, it is important to depict the overall trend in the variation of HAE prevalence, which will help provide evidence for preventive measures and HAE studies in the future. Therefore, this SR and subsequent meta-analysis aim to estimate the characteristics and trends in the variation of HAE prevalence in China and to explore potential influencing factors.

## Methods

### Search strategy and study selection

The SR and meta-analysis were performed according to the guidelines of PRISMA [19]. The online search was carried out using 6 databases (PubMed, Web Of Science, EMBASE, CNKI, Wanfang Data, and VIP) with keywords and Boolean operators AND/OR: “(alveolar echinococcosis OR alveolococcosis OR echinococcus multilocularis infection OR alveolar hydatid disease OR multilocular echinococcosis) AND (prevalence OR epidemiology) AND (human OR people OR person OR man OR men OR women OR woman OR patient) AND (China OR Chinese).” All the screened articles were published before December 1, 2019.

The study selection was performed independently by two researchers (XZW and GDD). Disagreements were resolved by consensus. First, we inspected the titles and abstracts of all the studies. Studies were included if they were cross-sectional studies, case-control studies, or cohort studies regarding HAE in China. Subsequently, we perused through the full text of all the articles. Studies were included if they used both imaging (B-ultrasonography) and serological examinations as diagnostic methods for HAE [20, 21]. Studies were excluded if they had been published repeatedly with the same samples, had inaccessible full texts, or had no data on HAE prevalence in China. We also identified relevant papers from the reference lists of the articles for the SR and meta-analysis.

### Data extraction and quality assessment

We used Microsoft Excel (version 2016) to record data that included the first author, published year, language, years during which the study was conducted, study areas, the sample size, number of HAE patients, and patient demographic details (sex, age, occupation) for each of the studies included. All the data were extracted independently by two researchers (XZW and ML). Disagreements were resolved by consensus.

The Agency for Healthcare Research and Quality (AHRQ) checklist [22] was used to evaluate the quality of the studies. Out of the 11 items in the AHRQ checklist, two items were unsuitable for these studies: item 4 (“Indicate whether or not subjects were consecutive if not population-based”) and item 11 (“Clarify what follow-up, if any, was expected and the percentage of patients for which incomplete data or follow-up was obtained”). Therefore, 9 items were used to score the studies (Additional file 1). If an item was answered with a “yes,” it was scored “1”; if it was answered with a “no or unclear,” it was scored “0.” The quality of the studies was classified using a number system, where low-quality studies scored 0–2, medium-quality studies scored 3–5, and high-quality studies scored 6–9.

### Statistical analysis

R software (version 3.6.1) was used for all analyses. The arcsine transformation was calculated for the prevalence of each study and the pooled prevalence of HAE was calculated as part of the meta-analysis. Heterogeneity was evaluated using the chi-square test and *I*^*2*^ statistic. If the *P*-value<0.10 and *I*^*2*^ ≥ 50%, substantial heterogeneity between studies was indicated, and the random-effects model was used [21]; otherwise, we used the fixed-effects model. The results of the meta-analysis were presented using forest plots, and publication bias of the studies was assessed by the Egger’s test and funnel plots. Subgroup analyses, sensitivity analyses, and meta-regression analyses were performed to analyze the source of heterogeneity and factors potentially influencing the prevalence of HAE.

### Ethical approval

Not applicable.

## Results

### Literature search results

**Fig. 1:**
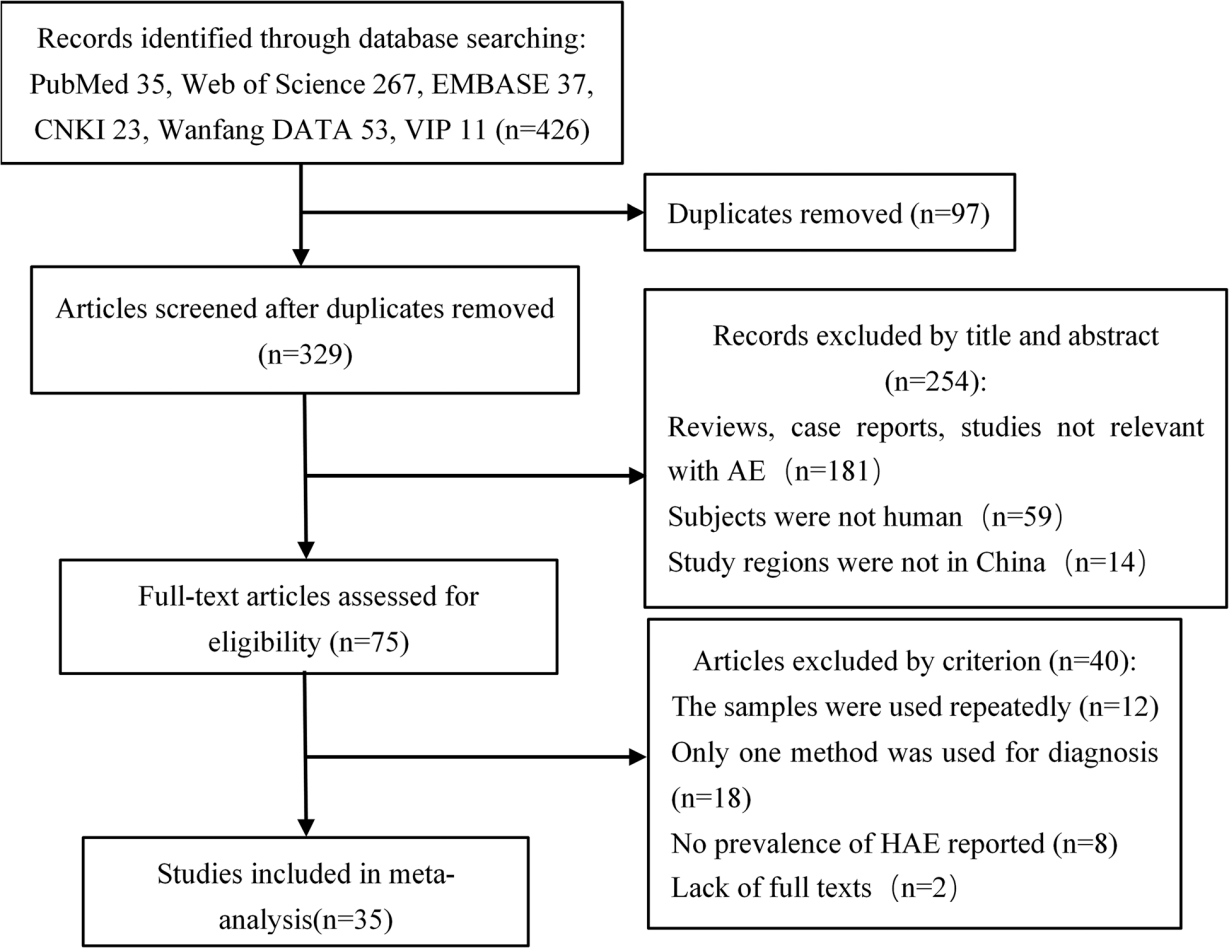
Flow diagram of the selection of studies

Based on the search strategy, 426 studies were considered, of which 87 were from Chinese databases. Ninety-seven studies that were duplicates were excluded by the document management software NoteExpress (version 3.2). After reading the titles and abstracts, 254 studies were excluded. The full texts of the remaining 75 papers were then screened by the selection criteria. A total of 35 eligible articles were included in the meta-analysis after screening, according to the inclusion and exclusion criteria. The details of the selection process are shown in Fig. 1. However, the study discussed in reference [7] was conducted across two periods (1997 and 2003). Therefore, we treated the data from the two periods as two different studies (reference 7-1, 7-2) in the meta-analysis. Similarly, the data for the study discussed in reference [23] were acquired from 6 areas (Gansu, Ningxia, Qinghai, Sichuan, Tibet, and Xinjiang); therefore, it was considered as 6 separate studies (reference 33-1, 33-2, 33-3, 33-4, 33-5, 33-6).

### Basic information of included literatures

Therefore, out of the 41 selected studies (after reference 7-1, 7-2 and reference 33-1, 33-2, 33-3, 33-4, 33-5, 33-6 were included), 11 (27%) were in English while 30 (73%) in Chinese (Table 1). The periods of time in which these studies were carried out and published were from 1991 to 2016 and from 1992 to 2019, respectively. All surveys were cross-sectional studies and were conducted in 6 provinces in China: 9 in Gansu, Ningxia (3), Qinghai (8), Sichuan (8), Tibet (9), and Xinjiang (4). Data on 2,032,811 subjects were included, of which 7522 were HAE patients. Table 1 shows the general features of the included studies.

**Table 1:** List of included articles in the meta-analysis

Study ID	Reference	Language	Conducted year	Study areas	Sample size	Cases	Score
1	[24]	English	1991	Gansu	1312	65	4
2	[25]	English	1997	Gansu	2482	84	6
3	[26]	Chinese	1998	Sichuan	3999	76	4
4	[27]	Chinese	2000	Qinghai	1046	3	3
5	[28]	English	1998	Sichuan	1858	43	2
6	[29]	English	1998	Qinghai	3703	29	5
7–1	[30]	Chinese	1997	Gansu	3116	113	2
7–2		Chinese	2003	Gansu	393	6	2
8	[31]	English	2002	Sichuan	3199	198	3
9	[32]	Chinese	2003	Sichuan	3018	37	3
10	[33]	English	2003	Sichuan	7138	223	3
11	[34]	Chinese	2005	Qinghai	6528	125	2
12	[35]	English	2002	Ningxia	159	8	3
13	[36]	English	2003	Ningxia	4773	96	5
14	[37]	Chinese	2005	Qinghai	1549	39	3
15	[38]	Chinese	2006	Qinghai	979	2	2
16	[39]	Chinese	2007	Qinghai	1723	141	3
17	[40]	Chinese	2007	Xinjiang	712	2	2
18	[41]	English	2008	Sichuan	10,186	311	3
19	[42]	Chinese	1997	Gansu	2485	86	3
20	[43]	Chinese	2009	Sichuan	538,208	4386	3
21	[44]	English	2007	Tibet	1511	11	2
22	[45]	Chinese	2008	Qinghai	1561	34	3
23	[46]	Chinese	2013	Xinjiang	532	2	4
24	[47]	Chinese	2011	Gansu	257,823	3	3
25	[48]	Chinese	2013	Xinjiang	42,356	2	3
26	[49]	Chinese	2015	Gansu	118,476	40	5
27	[50]	Chinese	2016	Tibet	10,287	3	4
28	[51]	Chinese	2016	Tibet	21,497	26	4
29	[52]	Chinese	2016	Tibet	77,049	136	5
30	[53]	Chinese	2016	Tibet	11,897	33	4
31	[54]	Chinese	2016	Tibet	14,289	39	5
32	[55]	Chinese	2016	Tibet	5016	5	5
33–1	[56]	Chinese	2016	Sichuan	112,605	301	6
33–2		Chinese	2016	Tibet	80,384	153	6
33–3		Chinese	2016	Gansu	198,131	10	6
33–4		Chinese	2016	Qinghai	109,122	573	6
33–5		Chinese	2016	Ningxia	62,348	13	6
33–6		Chinese	2016	Xinjiang	302,121	24	6
34	[57]	Chinese	2016	Tibet	4740	13	4
35	[58]	English	2006	Gansu	2500	28	4

### Literature quality evaluation

The quality of the cross-sectional studies was evaluated by the adjusted AHRQ checklist (9 items). The results (Table 1) showed that there were 7 low-quality studies (17%), 27 medium-quality studies (66%), and 7 high-quality studies (17%). The details of the quality evaluation were shown in Additional file 2.

### Meta-analysis of the prevalence of HAE in China

**Fig. 2:**
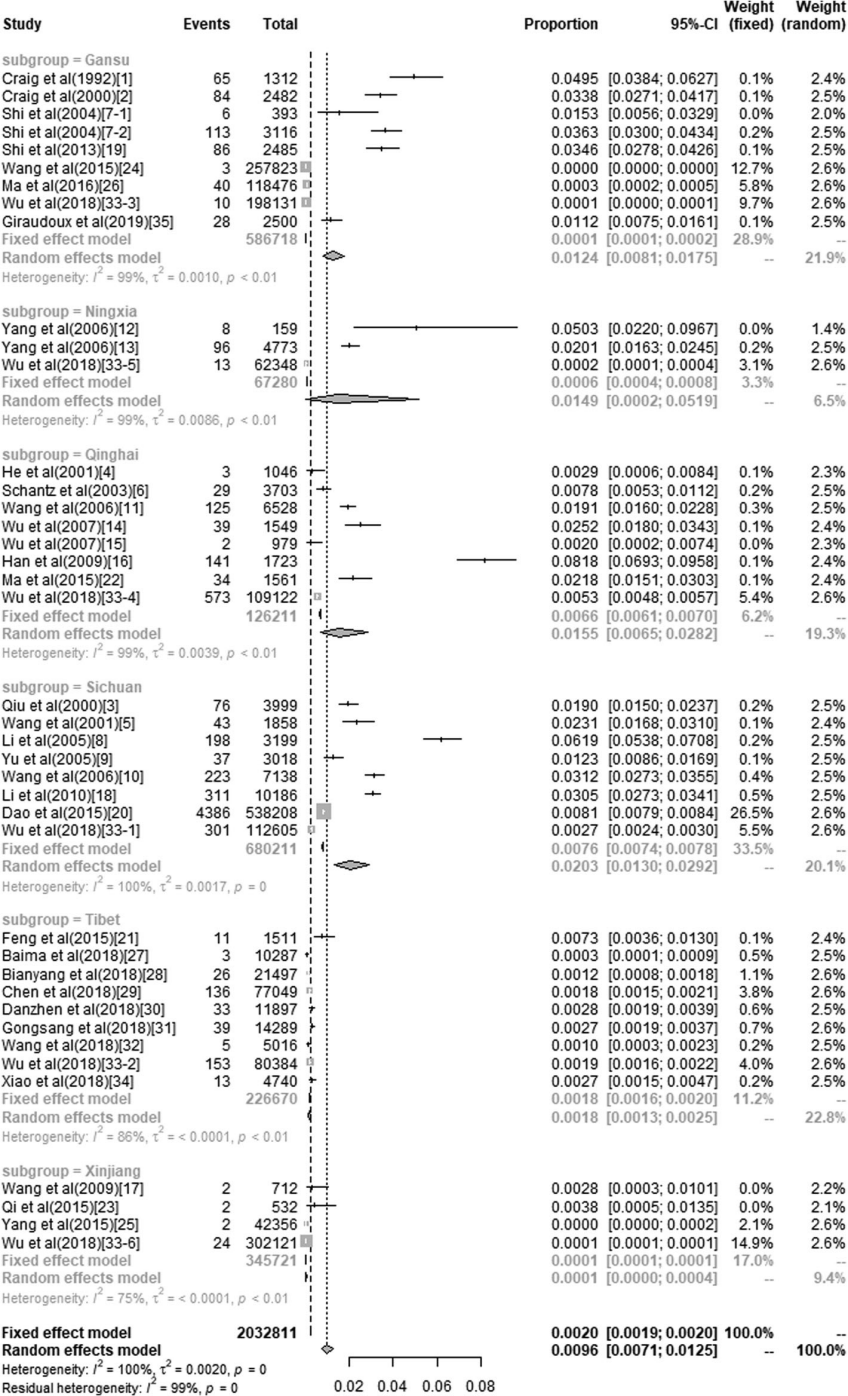
Forest plot of the meta-analysis for the pooled HAE prevalence in China

Substantial heterogeneity was observed among the included studies (*I*^*2*^ = 100%, *P* < 0.01, Fig. 2), which is common in most meta-analyses studying prevalence. Therefore, we used a random-effects model to calculate the pooled prevalence. The result indicated that the pooled prevalence of HAE in China was 0.96% (95% confidence interval [CI]: 0.71 to 1.25%) (Table 2). The funnel plot was shown in the Additional file 3 and the Egger’s test did not indicate any publication bias (*P* = 0.06). The sensitivity analysis indicated that despite excluding studies, the pooled prevalence remained stable (Additional file 4).

**Table 2:** Meta-analysis of HAE prevalence in China

Groups	Number of studies	Sample size	Prevalence,%(95% CI)	*I*^*2*^,%	*P* heterogeneity	*P* difference	Odds ratio(95% CI)	*P* Eggers’s test
Overall	41	2,032,811	0.96 (0.71–1.25)	100	<0.01			0.06
**Areas**						< 0.01		
Gansu	9	586,718	1.24 (0.81–1.75)	99	<0.01			–
Ningxia	3	67,280	1.49 (0.02–5.19)	99	<0.01			–
Qinghai	8	126,211	1.55 (0.65–2.82)	99	<0.01			–
Sichuan	8	680,211	2.03 (1.30–2.92)	100	<0.01			–
Tibet	9	226,670	0.19 (0.13–0.25)	86	<0.01			–
Xinjiang	4	345,721	0.013 (0.001–0.036)	75	<0.01			–
**Sex**						< 0.01		
female	11	22,595	3.47 (2.69–4.34)	91	<0.01		1.60 (1.35–1.91)	0.59
male	11	23,147	2.14 (1.55–2.82)	91	<0.01		–	0.98
**Age group**						< 0.01		
≥ 30 years	5	4720	6.41 (3.50–10.12)	95	<0.01		4.72 (2.29–9.75)	–
<30 years	5	6198	1.64 (0.36–3.83)	96	<0.01		–	–
**Occupation**						< 0.01		
farmer and/or herdsman	4	9987	2.90 (1.82–4.21)	90	<0.01		2.54 (1.60–4.02)	–
others	4	8777	1.20 (0.63–1.94)	86	<0.01		–	–

All the studies were divided into 6 groups based on the geographical areas for the subgroup analysis. The prevalence of HAE in these 6 areas were statistically significant (*P* < 0.01, Table 2). The highest prevalence of HAE was in Sichuan (2.03, 95% CI: 1.30 to 2.92%), while the lowest was in Xinjiang (0.013, 95% CI: 0.001 to 0.036%). There were not enough studies based in Xinjiang (4 studies) and Ningxia (3 studies), therefore, the pooled prevalence of these two areas may not be accurate.

### Factors potentially influencing HAE prevalence

Three factors potentially affecting HAE prevalence were considered in our meta-analysis: sex, age, and occupation. Due to the small number of articles addressing “age” and “occupation” (< 10 articles), publication bias was only evaluated for “sex.” Heterogeneity was indicated within all groups (sex, age, and occupation) (Additional file 5), with statistically significant differences observed (*P* < 0.01) (Table 2).

Of the included studies, 11 articles (1–3, 8–11, 13, 14, 18, 22) reported data on sex in their study populations. The meta-analysis showed that the prevalence of women with HAE (3.47, 95% CI: 2.69 to 4.34%) was higher than that of men with HAE (2.14, 95% CI: 1.55 to 2.82%) as shown in Table 2. The overall pooled odds ratio (OR) for women with HAE was 1.60 (95% CI: 1.35 to 1.91, *P*<0.01). After excluding reference [1] from the sensitivity analysis, we found that the heterogeneity reduced (*I*^*2*^ = 27%, *P* = 0.20), and the adjusted OR was 1.50 (95% CI: 1.30 to 1.73, *P*<0.01), as shown in Additional file 6. The funnel plot (Additional file 3) and Egger’s test did not indicate the existence of publication bias.

Five articles (1, 13, 14, 16, 22) had data on the age of the subjects. The prevalence of HAE was higher in those whose age was ≥30 years old (6.41, 95% CI: 3.50 to 10.12%) than those whose age was < 30 years (1.64, 95% CI: 0.36 to 3.83%) in Table 2. Being in the ≥30 years old age group was associated with an increase in HAE prevalence (OR = 4.72, 95% CI: 2.29 to 9.75, *P*<0.01). The sensitivity analysis (Additional file 7) indicated that the OR was relatively stable when any study was excluded.

The occupations of the subjects were investigated in 4 articles (3, 9, 18, and 22). The analysis showed that HAE prevalence was higher in farmers and/or herdsmen (2.90, 95% CI: 1.82 to 4.21%) than in people in other occupations (1.20, 95% CI: 0.63 to 1.94%), and the OR was 2.54 (95% CI: 1.60 to 4.02, *P*<0.01). The sensitivity analysis indicated that heterogeneity decreased (*I*^*2*^ = 50%, *P* = 0.14) when reference [22] was excluded; the adjusted OR was 3.01 (95% CI: 2.02 to 4.48, *P*<0.01) (Additional file 8).

### Trend in the variation of HAE prevalence in China

**Fig. 3:**
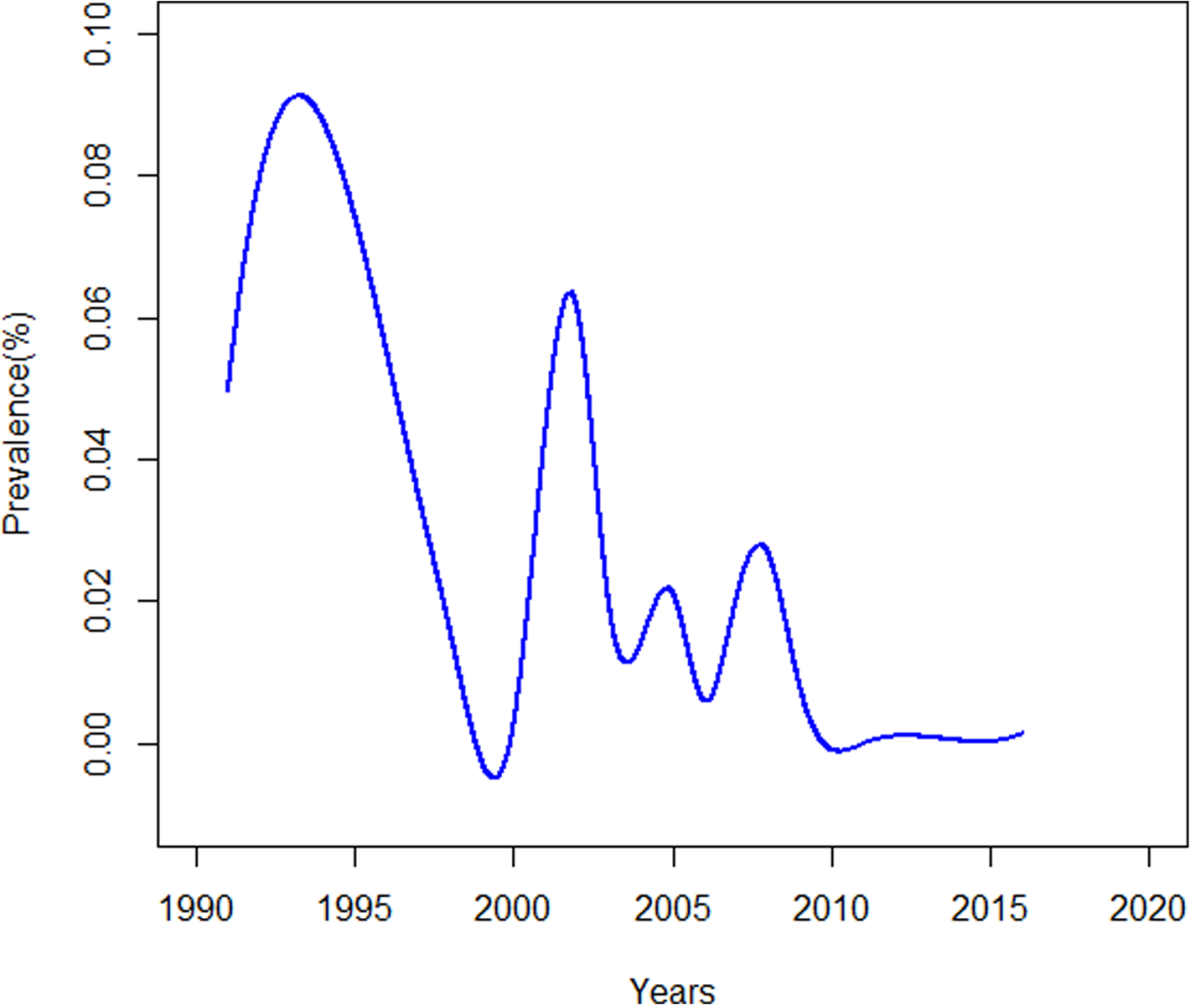
Trend in the variation of HAE prevalence in meta-analysis between 1991 and 2016

**Fig. 4:**
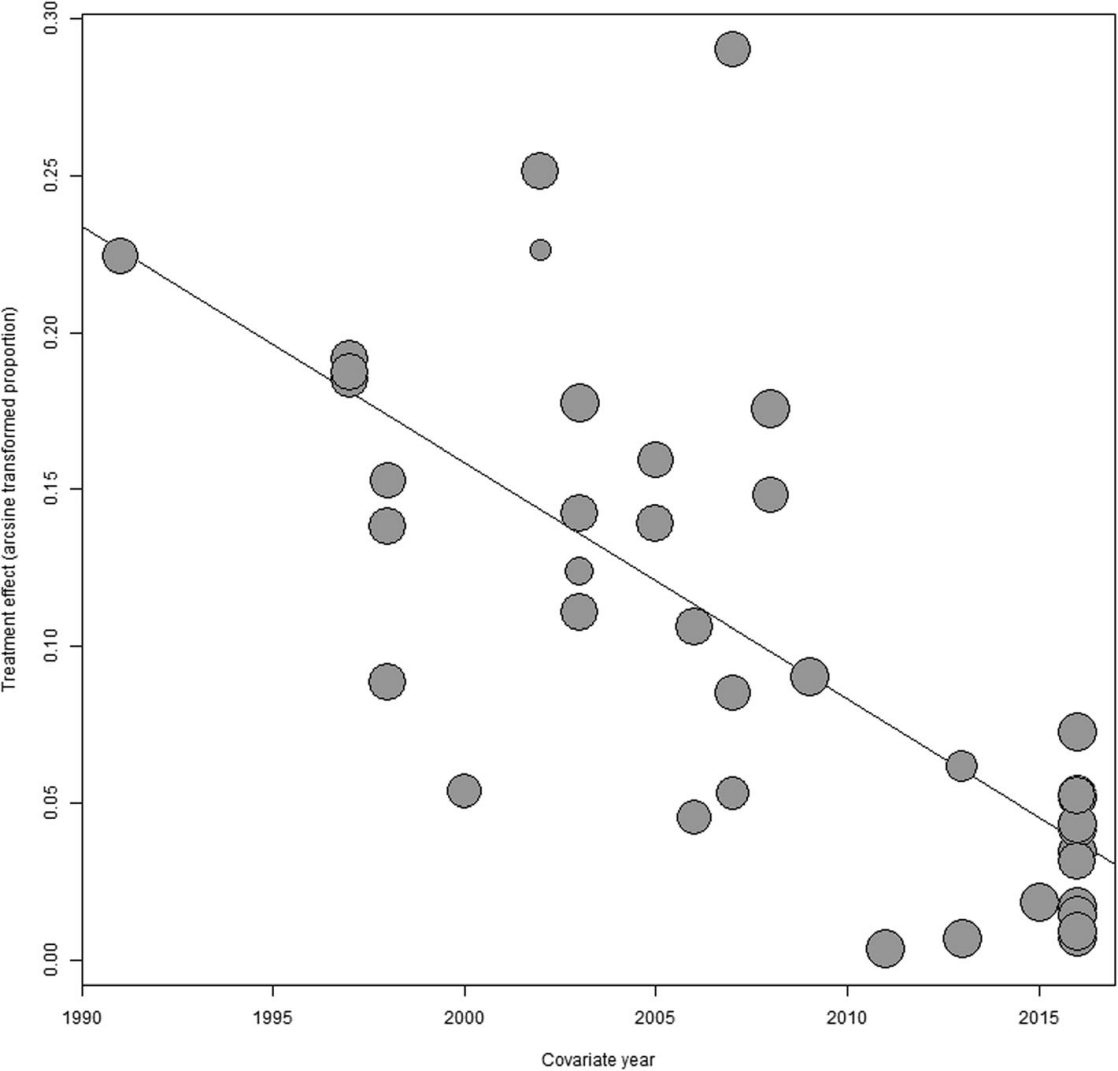
Meta-regression result of the time period during which the studies were conducted

Figure 3 shows the trend in the variation of HAE over time. The overall HAE prevalence has remained low since 2005—despite its resurgence in prevalence from 2005 to 2010, it remained stable after 2010. Additionally, the meta-regression analysis indicated that there was negative correlation between the period in which the studies were conducted and the HAE prevalence in China (*R*^2^ = 38.11%, *P* < 0.01) (Fig. 4).

## Discussion

This SR and meta-analysis included 41 studies published in 1992–2019, including 2,032,811 subjects and 7522 HAE patients. All included studies were cross-sectional, however, only seven were high-quality (17%). This means that researchers need to improve the quality of their studies for more reliable results. In the process of the literature search, we found that although researchers have carried out epidemiological investigations on HAE in recent years, there were very few nationwide epidemiological studies [56]. Therefore, this SR and meta-analysis summarized the HAE prevalence and provided epidemiological data about HAE in China for future prevention.

In China, we found that there were 6 provinces with high HAE prevalence (Table 2), which aligns with results from another study [10]. Moreover, of the 6 provinces, the prevalence in Sichuan and Qinghai were higher than in other provinces; since most of the included studies in Sichuan and Qinghai were conducted in communities residing in Tibetan autonomous prefectures, it is possible that the higher prevalence is related to the proximity of the Tibetan plateau [59]. The low-temperature climate caused by the high altitude in the Tibetan plateau makes survival easier for the *E. multilocularis* eggs in the environment, which is conducive to the spread of HAE [56]; additionally, certain religious beliefs (such as not killing dogs due to a respect for all animal life), and the limited hygiene practices (such as taking a bath once a year) of traditional Tibetan residents also allow for the spread of HAE [60]. However, the results of the meta-analysis indicated that the HAE prevalence in Tibet was relatively low. This might be because the Tibetan studies were conducted after 2010, when the national prevention and control project of echinococcosis resulted in increased advocacy in China. It is indicated that prevention measures should be implemented in the Tibetan plateau as well as the surrounding areas.

Female sex, being ≥30 years of age, and working as a farmer and/or herdsman were the three influencing factors identified in the context of HAE prevalence in our meta-analysis. Higher odds of HAE infection were observed in women when compared with men. These higher odds may be because women are responsible for feeding dogs in some Tibetan farming and pastoral areas, leading to frequent contact with a definitive host [61]. Women are also more involved in housework, such as collecting yak feces and sheep shearing. As a result, they are exposed to possible contaminants in dog feces and/or *E. multilocularis* eggs [33, 62]. Thus, specific living habits are a likely cause for the increase in the potential HAE risk in women. Five articles indicated that being ≥30 years of age increased the odds of an HAE infection. This may be because people older than 30 years of age have a greater chance of being infected [63, 64], and the long incubation period (5–15 years) of HAE can lead to a delayed onset of clinical symptoms [2, 65], which would make the disease more detectable in age groups ≥30 years. So it is important for concerned departments to focus on women and individuals older than 30 years for the prevention of HAE. Regarding occupation, the odds of HAE infection were higher in farmers and herdsmen than in people in other occupations. It is assumed that farmers and herdsmen are more easily exposed to dogs because of their occupation, which could increase the risk of infection. Yang et al. presented a similar result [48].

The sensitivity analysis indicated that reference [1] and reference [22] likely caused heterogeneity in the articles, addressing sex and occupations separately, possibly because their sample sizes were smaller than other studies in these articles. Compared to earlier years, the overall HAE prevalence has remained low since 2005, and there has been no indication that it has surged since 2010 (Fig. 3). However, it is necessary to keep monitoring levels through continuous surveillance and investigation in the future [8]. Additionally, the meta-regression analysis suggested that there was a negative correlation between the period in which the studies were conducted and the HAE prevalence, which corroborates the variation trend of HAE prevalence. A national echinococcosis control project has been carried out in China since 2005 [2], with new control programs and targets being formulated every 5 years since 2010 [23]. The measures of these programs were developed from three aspects: the population (screening and treatment), animals (dog management and deworming), and the environment (drinking water safety and rodent control). Based on the declining trend in HAE prevalence, it is proven that these prevention and control measures were effective and have resulted in the HAE prevalence being effectively controlled. Given the massive size of population, the magnitude of the susceptible population in China is still higher than in other countries [10, 56]. As a result of this, we should continue to implement One Health strategies that focus on the human control measures and establish a continuous surveillance system for HAE prevention [58, 66, 67].

In this SR and meta-analysis, we found that the prevalence of HAE in most studies was close to 0. This would have resulted in the confidence intervals spanning negative numbers, if the prevalence was directly used in this meta-analysis [68]. Therefore, the arcsine transformation was performed on the prevalence reported in each study to make the pooled results more reasonable. This method of data transformation also applies to other meta-analyses addressing disease prevalence. However, the literature included in this meta-analysis has substantial heterogeneity that may cause the pooled results to be inaccurate. Additionally, there was limited information available about HAE for data extraction, leading to insufficient information for the subgroup analysis.

## Conclusions

The prevalence of HAE varied in different areas in China. We found female sex, being ≥30 years of age, and working as a farmer and/or herdsman were the three influencing factors of HAE prevalence in our meta-analysis. It is important to focus on the vulnerable people with high risk of prevalence. We also described trends in the variation of HAE prevalence in China. HAE continues to pose a threat to public health in some regions of China, and several echinococcosis prevention programs utilizing One Health control strategies have been proposed to combat it. The One Health strategies emphasize interdisciplinary, cross-sectoral, and trans-regional cooperation as well as comprehensive control measures focused on the human-animal-environment relationship. Since One Health strategies were utilized for the preventive measures, HAE prevalence has been on a downward trend and has maintained low levels after 2010. Therefore, these One Health approaches should be used as reference points to formulate programs. High-quality epidemiological investigations and continuous surveillance programs ought to be conducted in the future in order to establish more measures for HAE control.

## Supplementary information

**Additional file 1.** The 9 items in adjusted AHRQ scale.

**Additional file 2.** Table of literature quality evaluation.

**Additional file 3.** Funnel plots of (a) overall studies (b) studies on male (c) studies on female.

**Additional file 4.** Sensitivity analysis of included articles.

**Additional file 5.** Forest plots of subgroup analyses.

**Additional file 6.** Meta-analysis for the potential influencing factor: sex.

**Additional file 7.** Meta-analysis for the potential influencing factor: age.

**Additional file 8.** Meta-analysis for the potential influencing factor: occupations.

## Data Availability

All the data associated with this article are included in the supplementary information files.

## Abbreviations

AE: Alveolar echinococcosis
PSCs: Protoscolices
HAE: Human alveolar echinococcosis
DALYs: Disability-adjusted life years
SR: Systematic review
AHRQ: Agency for Healthcare Research and Quality
OR: Odds ratio
CI: Confidence interval
