# Supplementary material for: Prevalence of human alveolar echinococcosis in China: a systematic review and meta-analysis
Source: BMC Public Health. 2020 Jul 14;20:1105. doi: 10.1186/s12889-020-08989-8 (PMC7362549; doi:10.1186/s12889-020-08989-8)
Supplement: Supplementary file 1 — Additional file 1. The 9 items in adjusted AHRQ scale. [file 12889_2020_8989_MOESM1_ESM.docx]

**Additional file 1** The 9 items in adjusted AHRQ scale

(1) Define the source of information (survey, record review)

(2) List inclusion and exclusion criteria for exposed and unexposed subjects (cases and controls) or refer to previous publications

(3) Indicate time period used for identifying patients

(4) Indicate if evaluators of subjective components of study were masked to other aspects of the status of the participants

(5) Describe any assessments undertaken for quality assurance purposes (e.g., test/retest of primary outcome measurements)

(6) Explain any patient exclusions from analysis

(7) Describe how confounding was assessed and/or controlled

(8) If applicable, explain how missing data were handled in the analysis

(9) Summarize patient response rates and completeness of data collection
