# Supplementary material for: Prevalence of human alveolar echinococcosis in China: a systematic review and meta-analysis
Source: BMC Public Health. 2020 Jul 14;20:1105. doi: 10.1186/s12889-020-08989-8 (PMC7362549; doi:10.1186/s12889-020-08989-8)
Supplement: Supplementary file 2 — Additional file 2. Table of literature quality evaluation. [file 12889_2020_8989_MOESM2_ESM.docx]

| **Additional file 2** Table of literature quality evaluation | | | | | | | | | | |
| --- | --- | --- | --- | --- | --- | --- | --- | --- | --- | --- |
| Study | Item (1) | Item (2) | Item (3) | Item (4) | Item (5) | Item (6) | Item (7) | Item (8) | Item (9) | Score |
| Craig et al(1992)[1] | 1 | 1 | 1 | 0 | 1 | 0 | 0 | 0 | 0 | 4 |
| Craig et al(2000)[2] | 1 | 1 | 1 | 0 | 1 | 0 | 1 | 0 | 1 | 6 |
| Qiu et al(2000)[3] | 1 | 1 | 1 | 0 | 0 | 0 | 1 | 0 | 0 | 4 |
| He et al(2001)[4] | 1 | 0 | 1 | 0 | 1 | 0 | 0 | 0 | 0 | 3 |
| Wang et al(2001)[5] | 1 | 0 | 1 | 0 | 0 | 0 | 0 | 0 | 0 | 2 |
| Schantz et al(2003)[6] | 1 | 1 | 1 | 0 | 0 | 1 | 1 | 0 | 0 | 5 |
| Shi et al(2004)[7-1] | 1 | 0 | 1 | 0 | 0 | 0 | 0 | 0 | 0 | 2 |
| Shi et al(2004)[7-2] | 1 | 0 | 1 | 0 | 0 | 0 | 0 | 0 | 0 | 2 |
| Li et al(2005)[8] | 1 | 1 | 1 | 0 | 0 | 0 | 0 | 0 | 0 | 3 |
| Yu et al(2005)[9] | 1 | 1 | 1 | 0 | 0 | 0 | 0 | 0 | 0 | 3 |
| Wang et al(2006)[10] | 1 | 1 | 1 | 0 | 0 | 0 | 0 | 0 | 0 | 3 |
| Wang et al(2006)[11] | 1 | 0 | 1 | 0 | 0 | 0 | 0 | 0 | 0 | 2 |
| Yang et al(2006)[12] | 1 | 1 | 1 | 0 | 0 | 0 | 0 | 0 | 0 | 3 |
| Yang et al(2006)[13] | 1 | 1 | 1 | 0 | 1 | 0 | 1 | 0 | 0 | 5 |
| Wu et al(2007)[14] | 1 | 1 | 1 | 0 | 0 | 0 | 0 | 0 | 0 | 3 |
| Wu et al(2007)[15] | 1 | 0 | 1 | 0 | 0 | 0 | 0 | 0 | 0 | 2 |
| Han et al(2009)[16] | 1 | 1 | 1 | 0 | 0 | 0 | 0 | 0 | 0 | 3 |
| Wang et al(2009)[17] | 1 | 0 | 1 | 0 | 0 | 0 | 0 | 0 | 0 | 2 |
| Li et al(2010)[18] | 1 | 1 | 1 | 0 | 0 | 0 | 0 | 0 | 0 | 3 |
| Shi et al(2013)[19] | 1 | 1 | 1 | 0 | 0 | 0 | 0 | 0 | 0 | 3 |
| Dao et al(2015)[20] | 1 | 0 | 1 | 0 | 0 | 0 | 1 | 0 | 0 | 3 |
| Feng et al(2015)[21] | 1 | 0 | 1 | 0 | 0 | 0 | 0 | 0 | 0 | 2 |
| Ma et al(2015)[22] | 1 | 1 | 1 | 0 | 0 | 0 | 0 | 0 | 0 | 3 |
| Qi et al(2015)[23] | 1 | 1 | 1 | 0 | 1 | 0 | 0 | 0 | 0 | 4 |
| Wang et al(2015)[24] | 1 | 1 | 1 | 0 | 0 | 0 | 0 | 0 | 0 | 3 |
| Yang et al(2015)[25] | 1 | 1 | 1 | 0 | 0 | 0 | 0 | 0 | 0 | 3 |
| Ma et al(2016)[26] | 1 | 1 | 1 | 0 | 1 | 0 | 1 | 0 | 0 | 5 |
| Baima et al(2018)[27] | 1 | 1 | 1 | 0 | 1 | 0 | 0 | 0 | 0 | 4 |
| Bianyang et al(2018)[28] | 1 | 1 | 1 | 0 | 1 | 0 | 0 | 0 | 0 | 4 |
| Chen et al(2018)[29] | 1 | 1 | 1 | 0 | 1 | 0 | 1 | 0 | 0 | 5 |
| Danzhen et al(2018)[30] | 1 | 1 | 1 | 0 | 1 | 0 | 0 | 0 | 0 | 4 |
| Gongsang et al(2018)[31] | 1 | 1 | 1 | 0 | 1 | 0 | 1 | 0 | 0 | 5 |
| Wang et al(2018)[32] | 1 | 1 | 1 | 0 | 1 | 0 | 1 | 0 | 0 | 5 |
| Wu et al(2018)[33-1] | 1 | 1 | 1 | 0 | 1 | 1 | 1 | 0 | 0 | 6 |
| Wu et al(2018)[33-2] | 1 | 1 | 1 | 0 | 1 | 1 | 1 | 0 | 0 | 6 |
| Wu et al(2018)[33-3] | 1 | 1 | 1 | 0 | 1 | 1 | 1 | 0 | 0 | 6 |
| Wu et al(2018)[33-4] | 1 | 1 | 1 | 0 | 1 | 1 | 1 | 0 | 0 | 6 |
| Wu et al(2018)[33-5] | 1 | 1 | 1 | 0 | 1 | 1 | 1 | 0 | 0 | 6 |
| Wu et al(2018)[33-6] | 1 | 1 | 1 | 0 | 1 | 1 | 1 | 0 | 0 | 6 |
| Xiao et al(2018)[34] | 1 | 1 | 1 | 0 | 1 | 0 | 0 | 0 | 0 | 4 |
| Giraudoux et al(2019)[35] | 1 | 1 | 1 | 0 | 0 | 0 | 1 | 0 | 0 | 4 |
