# Supplementary material for: Prevalence of human alveolar echinococcosis in China: a systematic review and meta-analysis
Source: BMC Public Health. 2020 Jul 14;20:1105. doi: 10.1186/s12889-020-08989-8 (PMC7362549; doi:10.1186/s12889-020-08989-8)

**Prevalence of human alveolar echinococcosis in China: a systematic review and meta-analysis**

Xuanzhuo Wang^1,2^· Guodong Dai^2^· Min Li^2^· Wanzhong Jia^2^· Zhongmin Guo^3*^· Jiahai Lu^1,4,5*^(ORCID: 0000-0002-8593-3402)

*Correspondence: Zhongmin Guo: 110822416@qq.com; Jiahai Lu: lujiahai@mail.sysu.edu.cn

1 School of Public Health, Sun Yat-sen University, Guangzhou, Guangdong Province, China

2 State Key Laboratory of Veterinary Etiological Biology, Key Laboratory of Veterinary Parasitology of Gansu Province, Lanzhou Veterinary Research Institute, Chinese Academy of Agricultural Sciences, Lanzhou, Gansu Province, China

3 Experimental Animal Center, Sun Yat-sen University, Guangzhou, Guangdong Province, China.

4 Key Laboratory for Tropical Diseases Control of Ministry of Education, Sun Yat-sen University, Guangzhou, Guangdong Province, China

5 One Health Research Centre, School of Public Health, Sun Yat-sen University, Guangzhou, Guangdong Province, China

**Supplementary file**

**Additional file 1** The 9 items in adjusted AHRQ scale

(1) Define the source of information (survey, record review)

(2) List inclusion and exclusion criteria for exposed and unexposed subjects (cases and controls) or refer to previous publications

(3) Indicate time period used for identifying patients

(4) Indicate if evaluators of subjective components of study were masked to other aspects of the status of the participants

(5) Describe any assessments undertaken for quality assurance purposes (e.g., test/retest of primary outcome measurements)

(6) Explain any patient exclusions from analysis

(7) Describe how confounding was assessed and/or controlled

(8) If applicable, explain how missing data were handled in the analysis

(9) Summarize patient response rates and completeness of data collection

| **Additional file 2** Table of literature quality evaluation | | | | | | | | | | |
| --- | --- | --- | --- | --- | --- | --- | --- | --- | --- | --- |
| Study | Item (1) | Item (2) | Item (3) | Item (4) | Item (5) | Item (6) | Item (7) | Item (8) | Item (9) | Score |
| Craig et al(1992)[1] | 1 | 1 | 1 | 0 | 1 | 0 | 0 | 0 | 0 | 4 |
| Craig et al(2000)[2] | 1 | 1 | 1 | 0 | 1 | 0 | 1 | 0 | 1 | 6 |
| Qiu et al(2000)[3] | 1 | 1 | 1 | 0 | 0 | 0 | 1 | 0 | 0 | 4 |
| He et al(2001)[4] | 1 | 0 | 1 | 0 | 1 | 0 | 0 | 0 | 0 | 3 |
| Wang et al(2001)[5] | 1 | 0 | 1 | 0 | 0 | 0 | 0 | 0 | 0 | 2 |
| Schantz et al(2003)[6] | 1 | 1 | 1 | 0 | 0 | 1 | 1 | 0 | 0 | 5 |
| Shi et al(2004)[7-1] | 1 | 0 | 1 | 0 | 0 | 0 | 0 | 0 | 0 | 2 |
| Shi et al(2004)[7-2] | 1 | 0 | 1 | 0 | 0 | 0 | 0 | 0 | 0 | 2 |
| Li et al(2005)[8] | 1 | 1 | 1 | 0 | 0 | 0 | 0 | 0 | 0 | 3 |
| Yu et al(2005)[9] | 1 | 1 | 1 | 0 | 0 | 0 | 0 | 0 | 0 | 3 |
| Wang et al(2006)[10] | 1 | 1 | 1 | 0 | 0 | 0 | 0 | 0 | 0 | 3 |
| Wang et al(2006)[11] | 1 | 0 | 1 | 0 | 0 | 0 | 0 | 0 | 0 | 2 |
| Yang et al(2006)[12] | 1 | 1 | 1 | 0 | 0 | 0 | 0 | 0 | 0 | 3 |
| Yang et al(2006)[13] | 1 | 1 | 1 | 0 | 1 | 0 | 1 | 0 | 0 | 5 |
| Wu et al(2007)[14] | 1 | 1 | 1 | 0 | 0 | 0 | 0 | 0 | 0 | 3 |
| Wu et al(2007)[15] | 1 | 0 | 1 | 0 | 0 | 0 | 0 | 0 | 0 | 2 |
| Han et al(2009)[16] | 1 | 1 | 1 | 0 | 0 | 0 | 0 | 0 | 0 | 3 |
| Wang et al(2009)[17] | 1 | 0 | 1 | 0 | 0 | 0 | 0 | 0 | 0 | 2 |
| Li et al(2010)[18] | 1 | 1 | 1 | 0 | 0 | 0 | 0 | 0 | 0 | 3 |
| Shi et al(2013)[19] | 1 | 1 | 1 | 0 | 0 | 0 | 0 | 0 | 0 | 3 |
| Dao et al(2015)[20] | 1 | 0 | 1 | 0 | 0 | 0 | 1 | 0 | 0 | 3 |
| Feng et al(2015)[21] | 1 | 0 | 1 | 0 | 0 | 0 | 0 | 0 | 0 | 2 |
| Ma et al(2015)[22] | 1 | 1 | 1 | 0 | 0 | 0 | 0 | 0 | 0 | 3 |
| Qi et al(2015)[23] | 1 | 1 | 1 | 0 | 1 | 0 | 0 | 0 | 0 | 4 |
| Wang et al(2015)[24] | 1 | 1 | 1 | 0 | 0 | 0 | 0 | 0 | 0 | 3 |
| Yang et al(2015)[25] | 1 | 1 | 1 | 0 | 0 | 0 | 0 | 0 | 0 | 3 |
| Ma et al(2016)[26] | 1 | 1 | 1 | 0 | 1 | 0 | 1 | 0 | 0 | 5 |
| Baima et al(2018)[27] | 1 | 1 | 1 | 0 | 1 | 0 | 0 | 0 | 0 | 4 |
| Bianyang et al(2018)[28] | 1 | 1 | 1 | 0 | 1 | 0 | 0 | 0 | 0 | 4 |
| Chen et al(2018)[29] | 1 | 1 | 1 | 0 | 1 | 0 | 1 | 0 | 0 | 5 |
| Danzhen et al(2018)[30] | 1 | 1 | 1 | 0 | 1 | 0 | 0 | 0 | 0 | 4 |
| Gongsang et al(2018)[31] | 1 | 1 | 1 | 0 | 1 | 0 | 1 | 0 | 0 | 5 |
| Wang et al(2018)[32] | 1 | 1 | 1 | 0 | 1 | 0 | 1 | 0 | 0 | 5 |
| Wu et al(2018)[33-1] | 1 | 1 | 1 | 0 | 1 | 1 | 1 | 0 | 0 | 6 |
| Wu et al(2018)[33-2] | 1 | 1 | 1 | 0 | 1 | 1 | 1 | 0 | 0 | 6 |
| Wu et al(2018)[33-3] | 1 | 1 | 1 | 0 | 1 | 1 | 1 | 0 | 0 | 6 |
| Wu et al(2018)[33-4] | 1 | 1 | 1 | 0 | 1 | 1 | 1 | 0 | 0 | 6 |
| Wu et al(2018)[33-5] | 1 | 1 | 1 | 0 | 1 | 1 | 1 | 0 | 0 | 6 |
| Wu et al(2018)[33-6] | 1 | 1 | 1 | 0 | 1 | 1 | 1 | 0 | 0 | 6 |
| Xiao et al(2018)[34] | 1 | 1 | 1 | 0 | 1 | 0 | 0 | 0 | 0 | 4 |
| Giraudoux et al(2019)[35] | 1 | 1 | 1 | 0 | 0 | 0 | 1 | 0 | 0 | 4 |


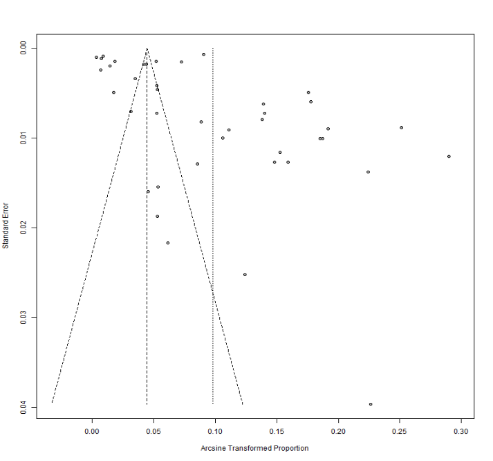


a


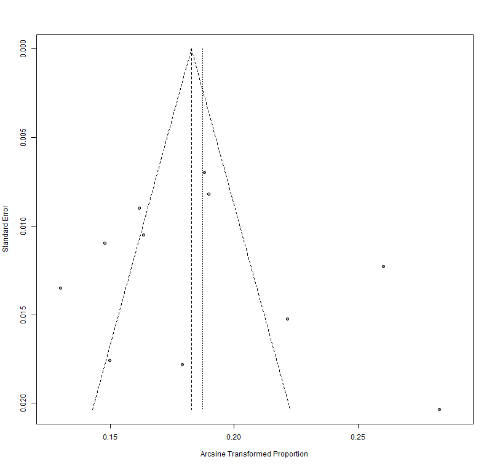


b


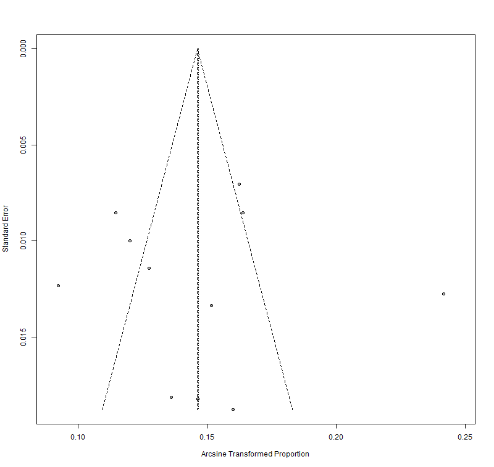


c

**Additional file 3** Funnel plots of (a) overall studies (b) studies on male (c) studies on female

age


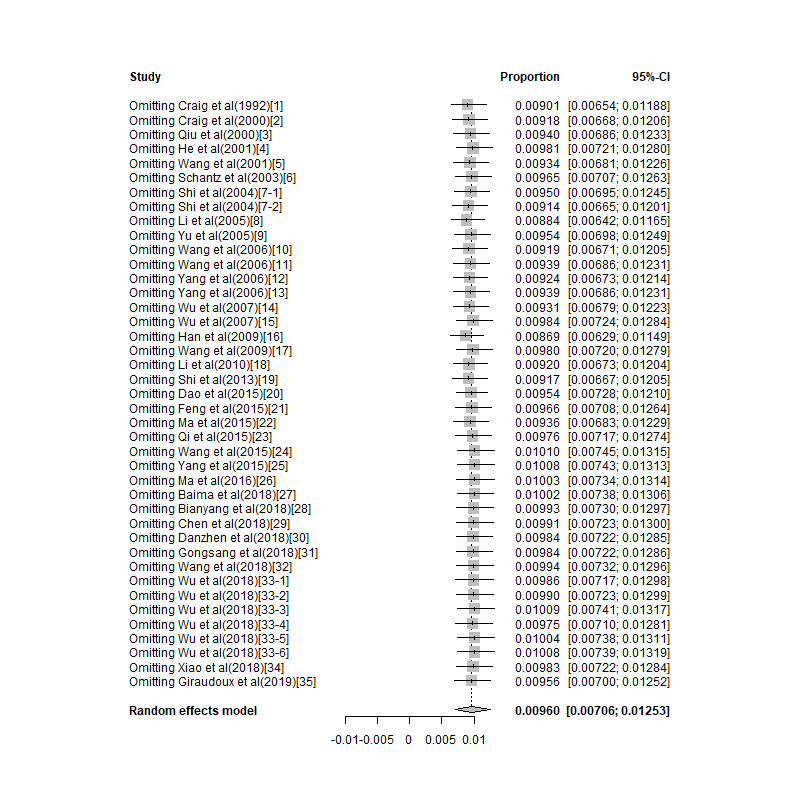


**Additional file 4** Sensitivity analysis of included articles


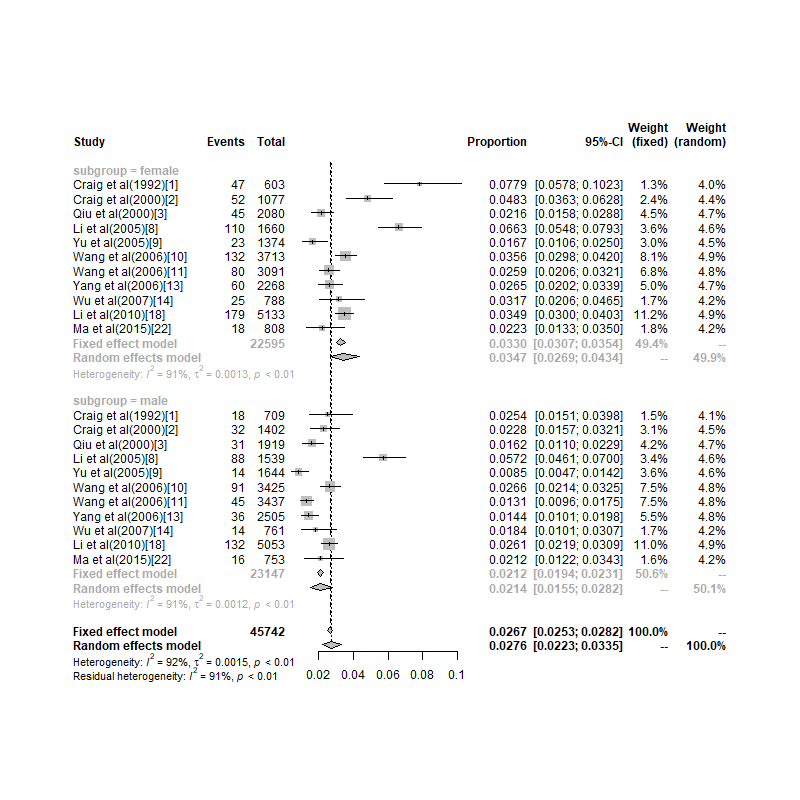


a


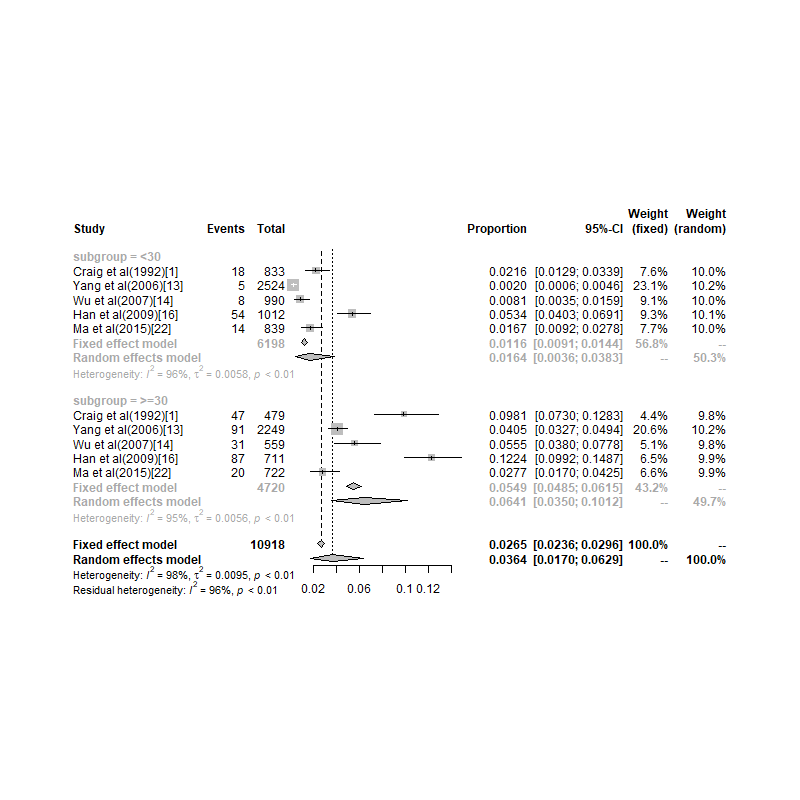


b

**Additional file 5** Forest plots of subgroup analyses (a: sex b: age groups c: occupations)

c


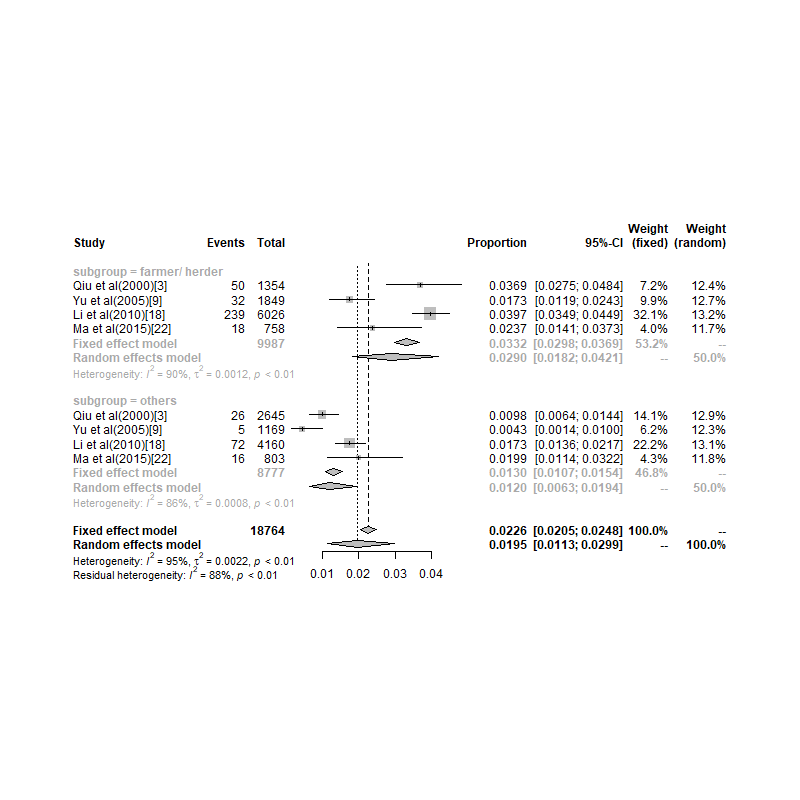

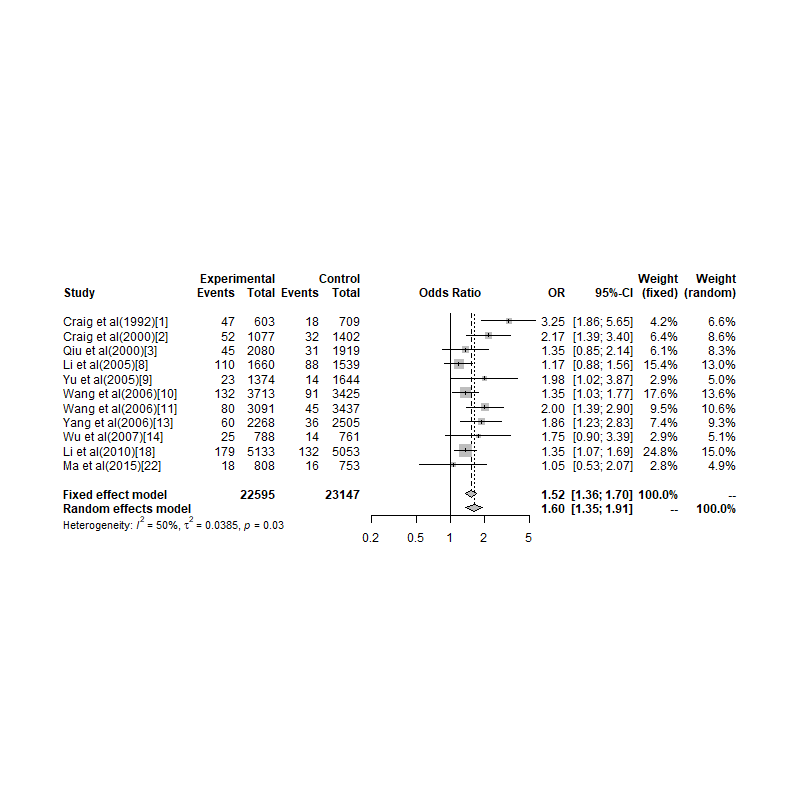


a


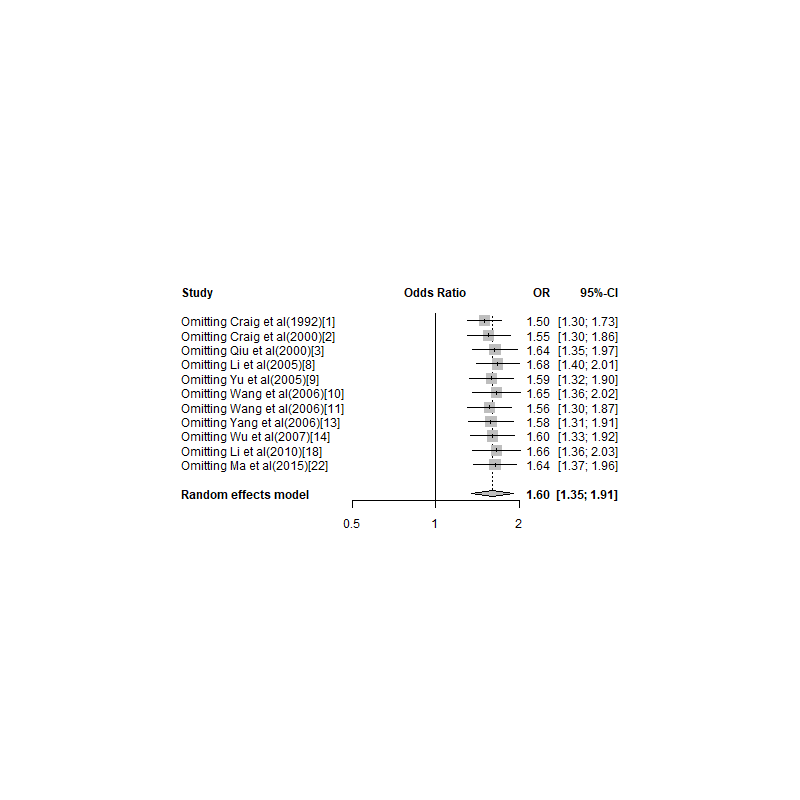


b


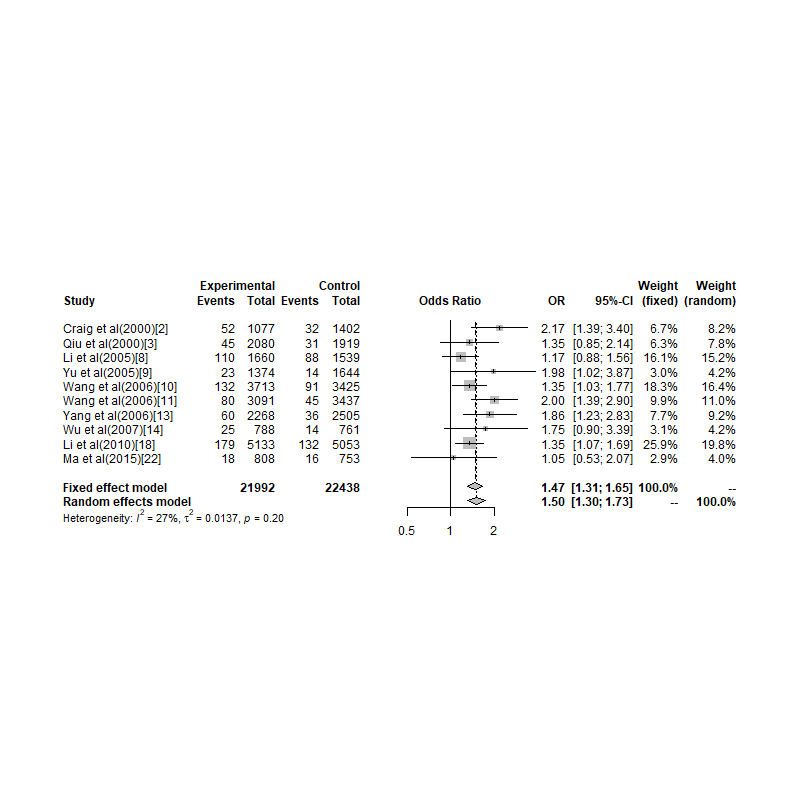


c

**Additional file 6** Meta-analysis for the potential influencing factor: sex (a: the forest plot of OR b: sensitivity analysis c: forest plot of adjusted OR)

**Additional file 7** Meta-analysis for the potential influencing factor: age (a: the forest plot of OR b: sensitivity analysis)

a


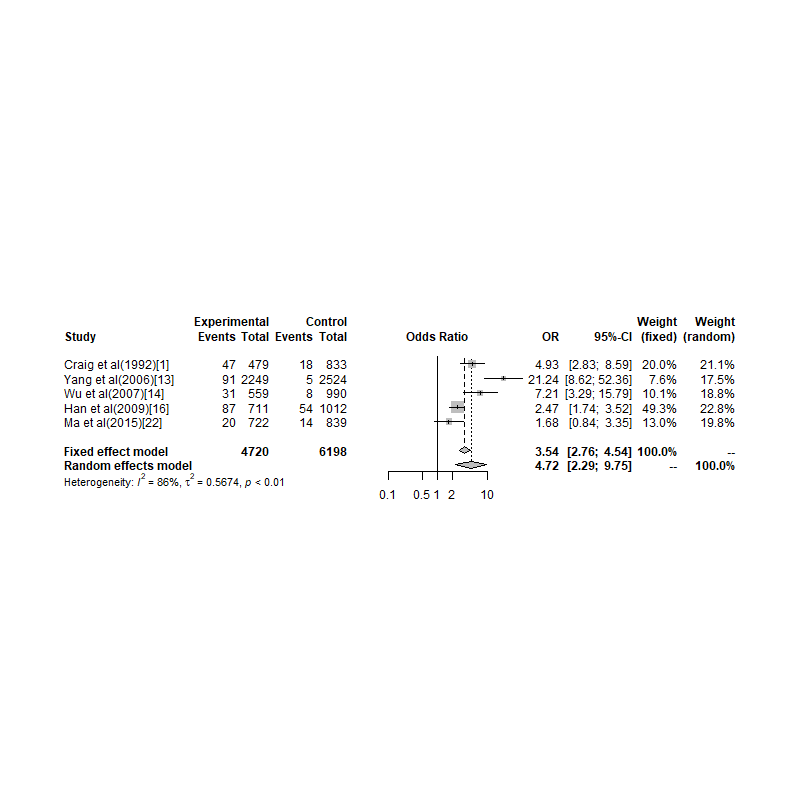


b


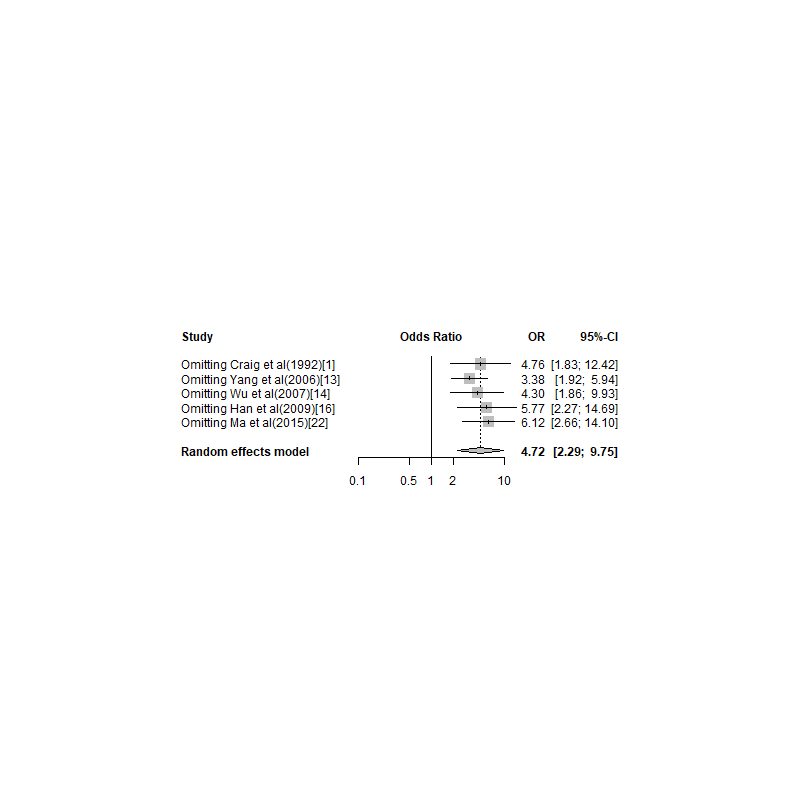


**Additional file 8** Meta-analysis for the potential influencing factor: occupations (a: the forest plot of OR b: sensitivity analysis c: forest plot of adjusted OR)

a


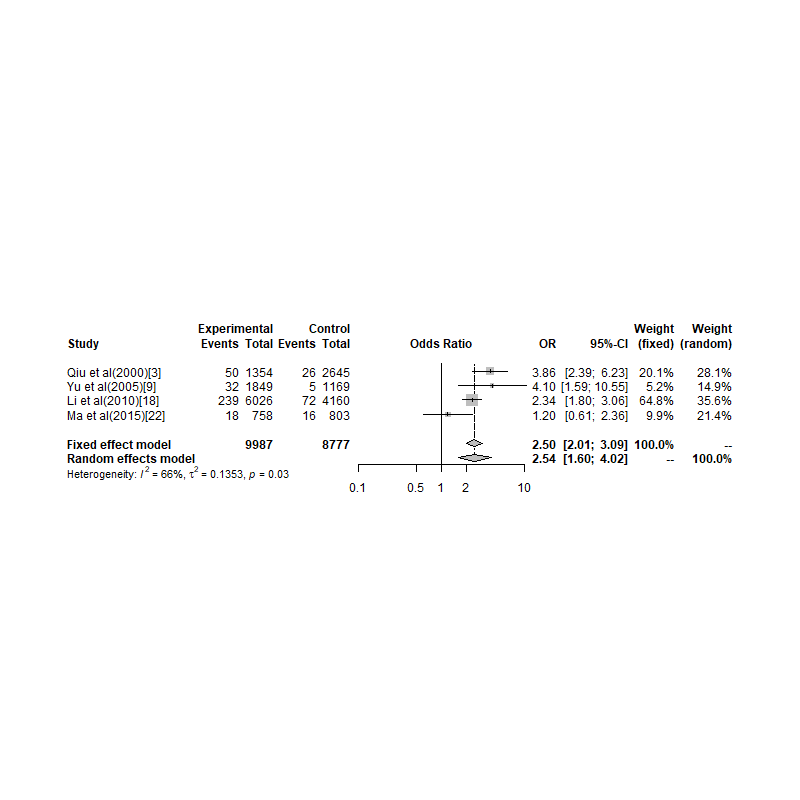


b


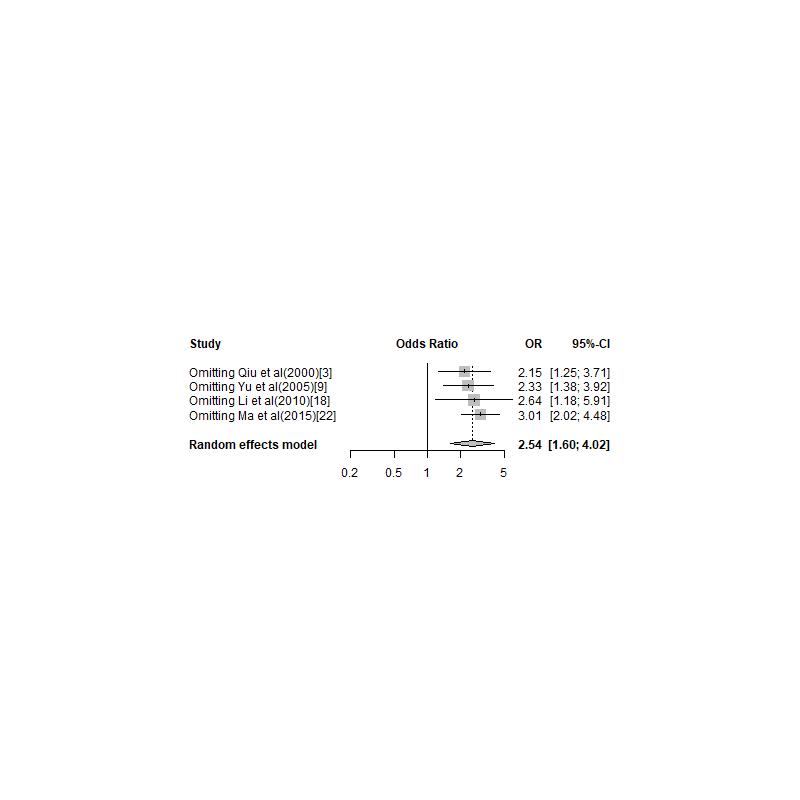


c


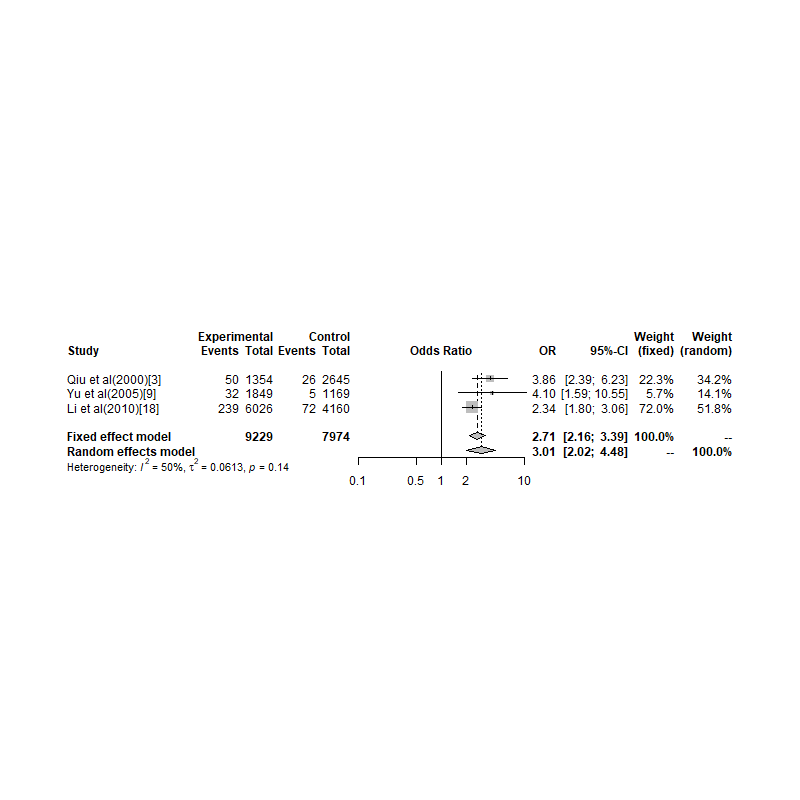

Supplement: Supplementary file 3 — Additional file 3. Funnel plots of (a) overall studies (b) studies on male (c) studies on female. [file 12889_2020_8989_MOESM3_ESM.docx]
