# Supplementary figures and images for: Prevalence of human alveolar echinococcosis in China: a systematic review and meta-analysis
Source: BMC Public Health. 2020 Jul 14;20:1105. doi: 10.1186/s12889-020-08989-8 (PMC7362549; doi:10.1186/s12889-020-08989-8)

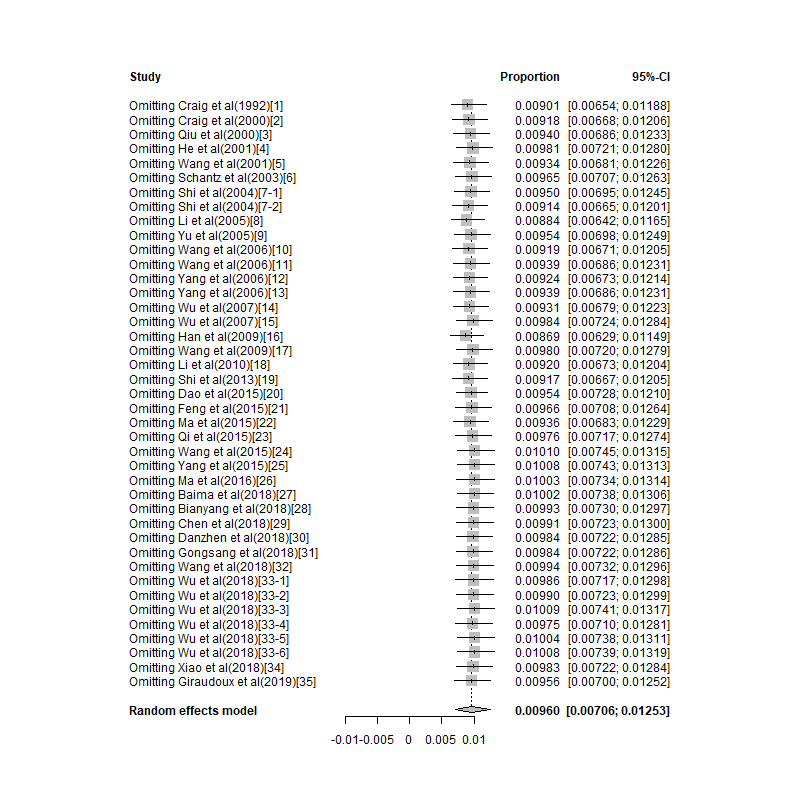


**Additional file 4** Sensitivity analysis of included articles

Supplement: Supplementary file 4 — Additional file 4. Sensitivity analysis of included articles. [file 12889_2020_8989_MOESM4_ESM.docx]

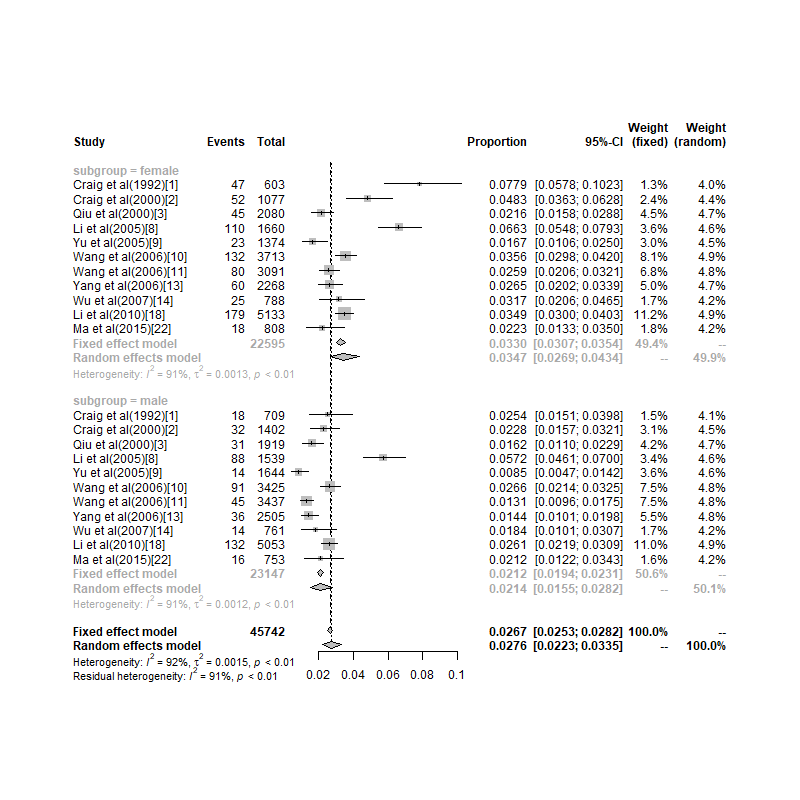


a


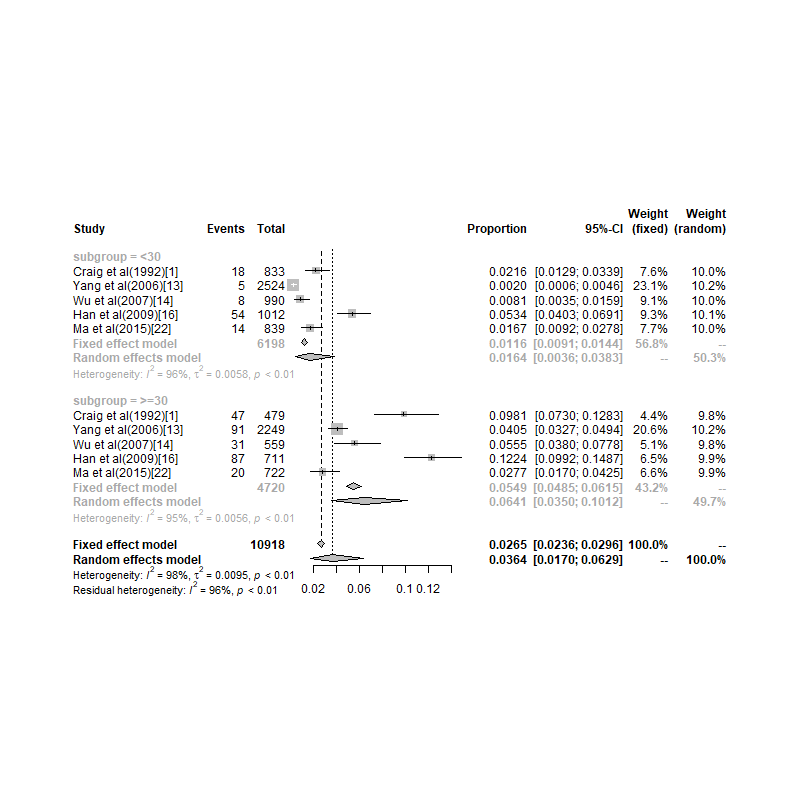


b

c


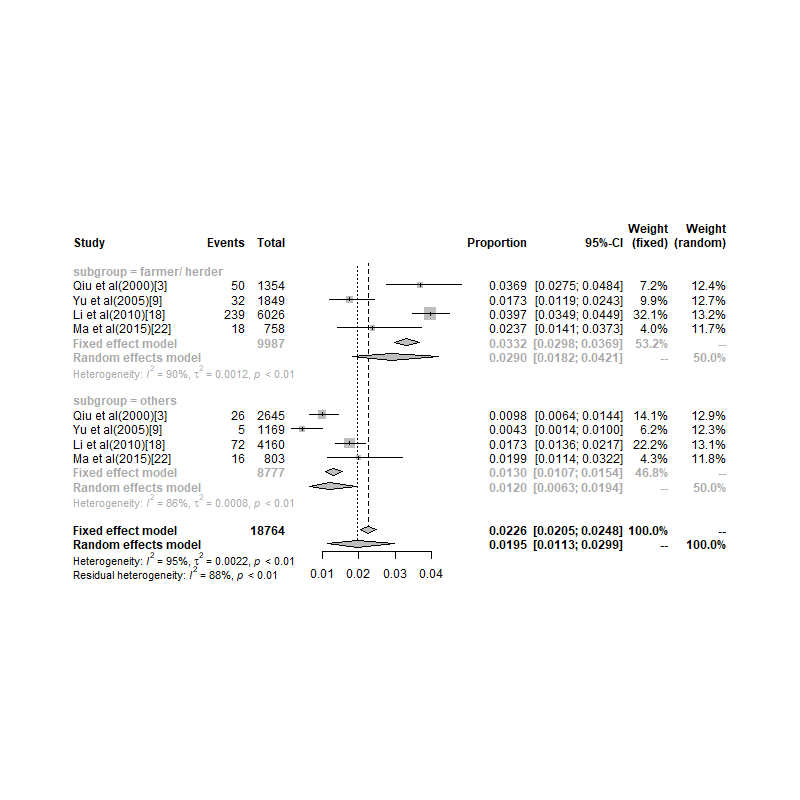


**Additional file 5** Forest plots of subgroup analyses (a: sex b: age groups c: occupations)

Supplement: Supplementary file 5 — Additional file 5. Forest plots of subgroup analyses. [file 12889_2020_8989_MOESM5_ESM.docx]
