# Supplementary material for: Prevalence of human alveolar echinococcosis in China: a systematic review and meta-analysis
Source: BMC Public Health. 2020 Jul 14;20:1105. doi: 10.1186/s12889-020-08989-8 (PMC7362549; doi:10.1186/s12889-020-08989-8)

**Additional file 7** Meta-analysis for the potential influencing factor: age (a: the forest plot of OR b: sensitivity analysis)

a


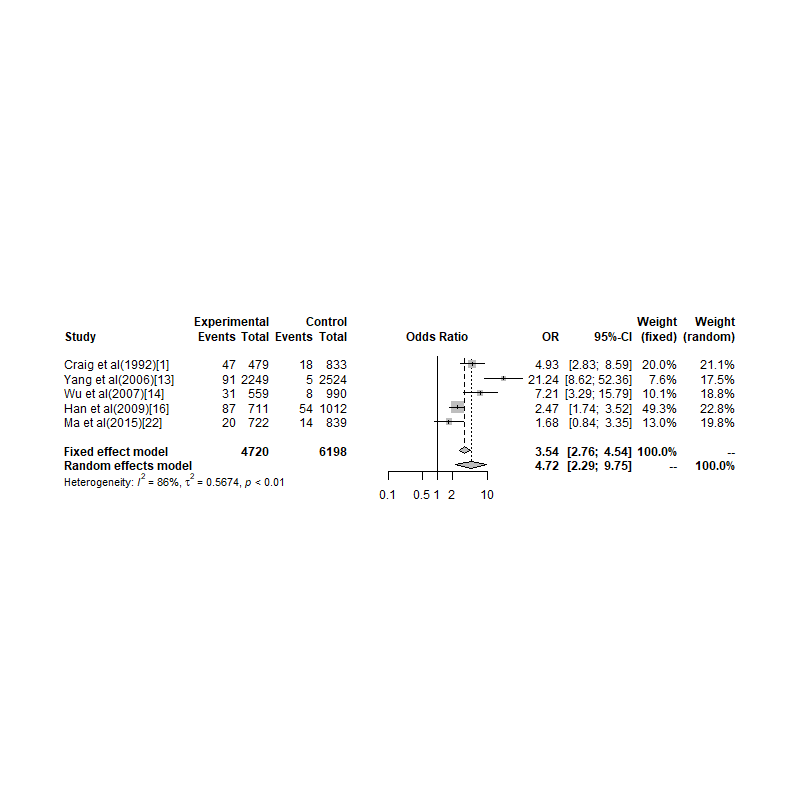


b


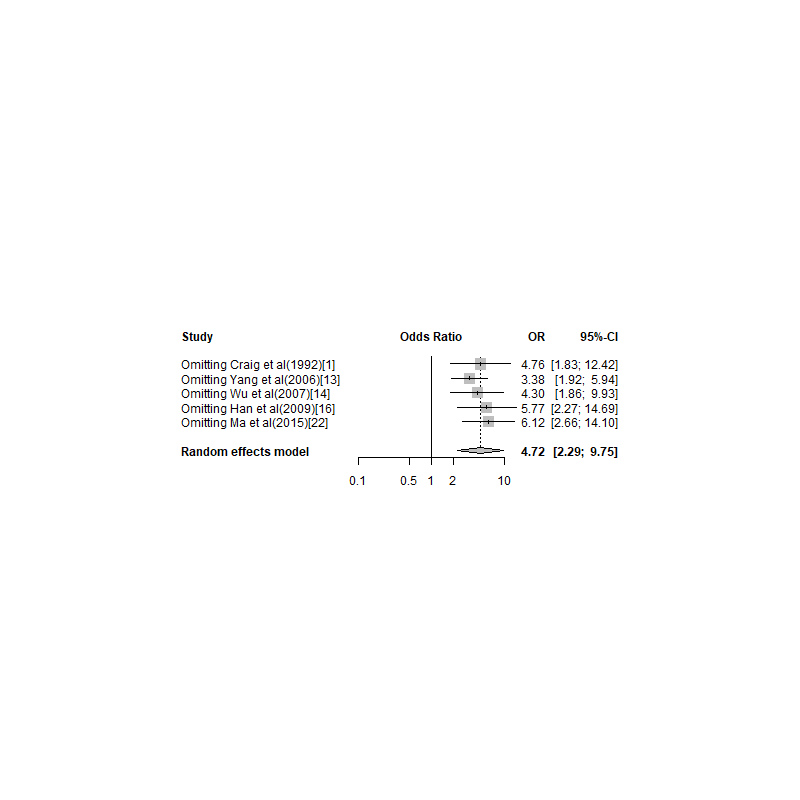

Supplement: Supplementary file 7 — Additional file 7. Meta-analysis for the potential influencing factor: age. [file 12889_2020_8989_MOESM7_ESM.docx]
