# Supplementary material for: Prevalence of human alveolar echinococcosis in China: a systematic review and meta-analysis
Source: BMC Public Health. 2020 Jul 14;20:1105. doi: 10.1186/s12889-020-08989-8 (PMC7362549; doi:10.1186/s12889-020-08989-8)

**Additional file 8** Meta-analysis for the potential influencing factor: occupations (a: the forest plot of OR b: sensitivity analysis c: forest plot of adjusted OR)

a


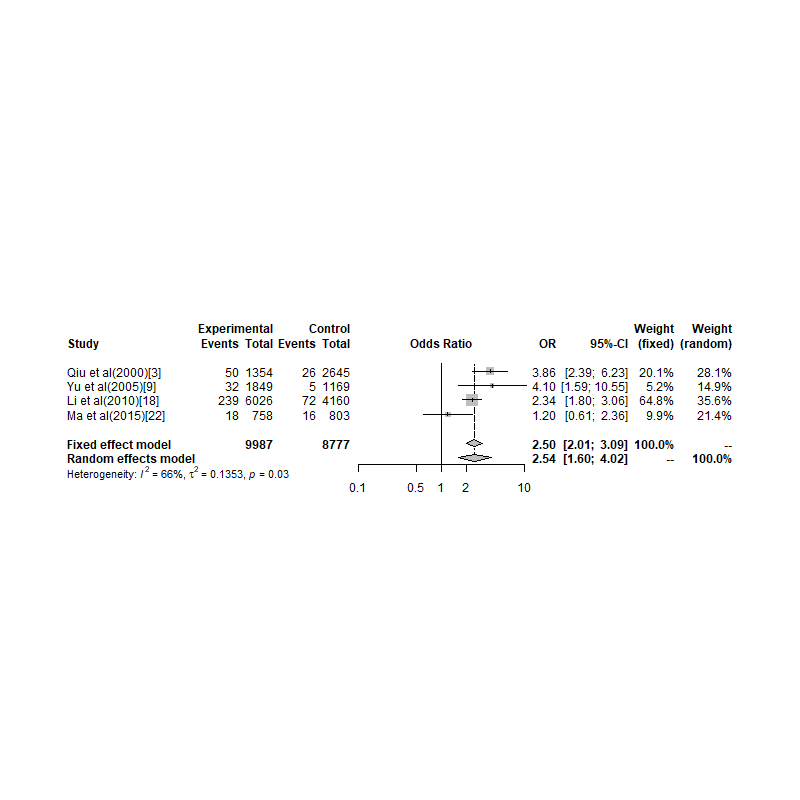


b


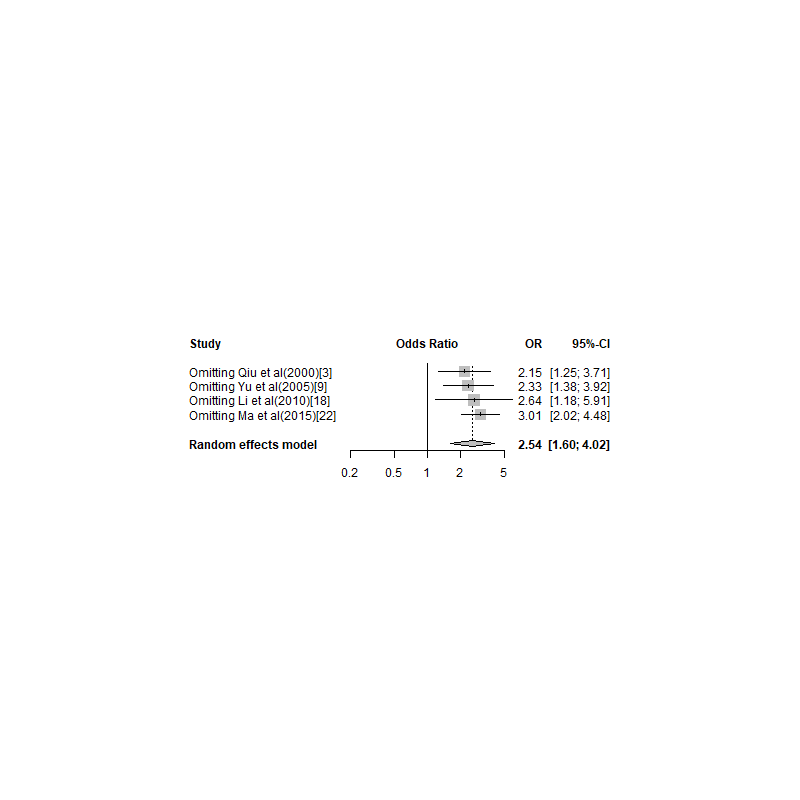


c


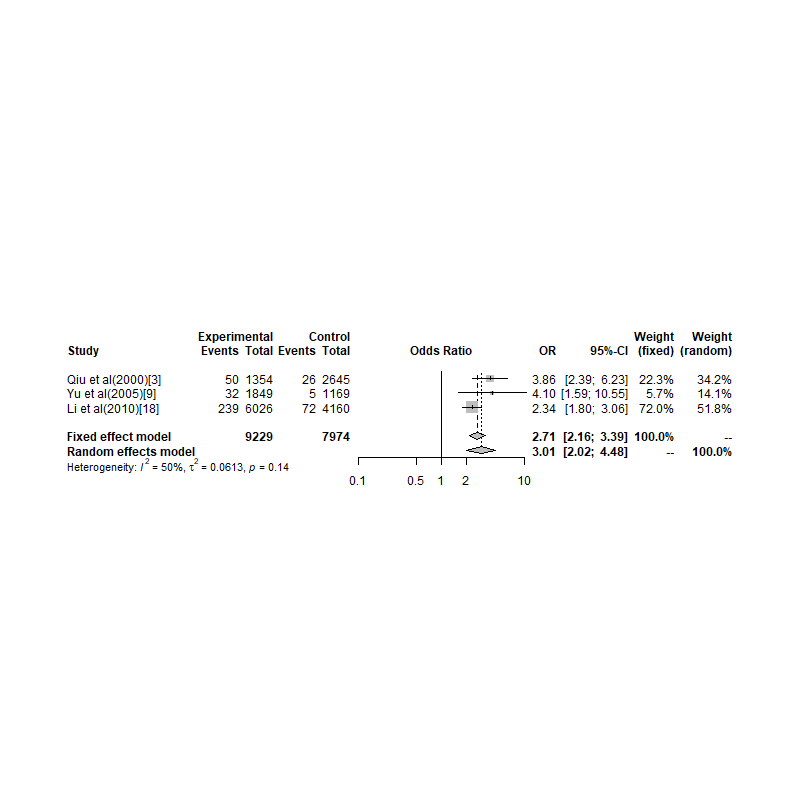

Supplement: Supplementary file 8 — Additional file 8. Meta-analysis for the potential influencing factor: occupations. [file 12889_2020_8989_MOESM8_ESM.docx]
